# Bovine NMRAL2 Protein Blunts Nitric Oxide Production and Inflammatory Response in *Mycobacterium bovis* Infected Bovine Lung Epithelial Cells

**DOI:** 10.3390/cells13231953

**Published:** 2024-11-24

**Authors:** Yongchong Peng, Shiying Zhou, Qin Sun, Xinjun Zhou, Chao Wang, Zijian Wang, Tahira Iftakhar, Yifan Zhu, Shengsong Xie, Xi Chen, Lei Zhang, Changmin Hu, Yingyu Chen, Aizhen Guo

**Affiliations:** 1 National Key Laboratory of Agricultural Microbiology, College of Veterinary Medicine, Huazhong Agricultural University, Wuhan 430070, China; ycpeng@mail.hzau.edu.cn (Y.P.);; 2 National Professional Laboratory for Animal Tuberculosis (Wuhan) of Ministry of Agriculture and Rural Affairs, International Research Center for Animal Disease, Ministry of Science and Technology, Huazhong Agricultural University, Wuhan 430070, China

**Keywords:** *Mycobacterium bovis*, NmrA-like gene, bovine, nitric oxide, inflammatory response, tuberculosis

## Abstract

Tuberculosis (TB), primarily caused by *Mycobacterium tuberculosis* (*M. tb*) and *Mycobacterium bovis* (*M. bovis*), remains the leading cause of death from a single infectious agent globally. Intracellular survival is crucial for their virulence; yet, the underlying mechanisms are not fully understood. This study aimed to demonstrate the significance of a previously unannotated bovine gene ENSBTAG00000011305 in *M. bovis* intracellular survival. This gene was termed *NMRAL2_Bovine* due to its inclusion of the NmrA domain which has a relation to nitric oxide (NO) production. We used CRISPR/Cas9 to knock out *NMRAL2_Bovine* in bovine lung epithelial cells and observed a significant decrease in *M. bovis*-induced cell death and the intracellular bacterial count, alongside increased NO levels. A transcriptome analysis revealed the upregulation of pathways linked to NO, IL-6, and TNF-α production, which was confirmed by the increased expression of iNOS, IL-6, and TNF-α. Correspondingly, Western blotting indicated that key signaling pathways, including NF-κB and MAPK, were activated. In conclusion, our findings determined that NMRAL2_Bovine functions as a negative regulator of the inflammatory response induced by *M. bovis* infection at the cellular level and, thereby, provide a novel insight into TB pathogenesis and a potential target for developing novel host-directed therapies against TB.

## 1. Introduction

Tuberculosis (TB), primarily caused by *Mycobacterium tuberculosis* (*M. tb*) and *Mycobacterium bovis* (*M. bovis*), is a leading cause of death from a single infectious agent worldwide [1], remaining a global health threat, and *M. bovis* contributes to approximately 10% of human TB cases. This situation is exacerbated by the limitations of early detection, the limited efficacy of the *Bacille Calmette–Guerin* (BCG) vaccine, derived from an attenuated strain of *M. bovis*, and the emergence of drug-resistant strains [2,3,4]. These issues often arise from an incomplete understanding of TB pathogenesis.

As a class of obligate intracellular bacteria, both *M. tb* and *M. bovis* has developed complicated strategies to evade host immune responses, utilizing different host factors to establish and sustain infection. Thus, understanding these interactions is vital for unraveling the complexities of TB pathogenesis.

Nitric oxide (NO) is an essential signaling molecule that plays a crucial role in the immune response against TB [5,6]. It activates the immune response of the host to eliminate intracellular *M. tb*. NO production is an essential aspect of the innate immune response against TB, promoting the destruction of pathogens by generating reactive nitrogen species (RNS) that directly damage bacterial components and inhibit their replication. However, maintaining an appropriate level of NO is critical, as excessive production can lead to tissue damage and inflammation, complicating the disease outcome [7,8,9,10,11,12,13].

Historically, NmrA-like proteins have been discovered in some fungi and bacteria, including PadA from *Dictyostelium discoideum*, PcNMRAL1 from *Phytophthora capsica*, and QOR2 from *Escherichia coli* [14,15,16]. In mammals, an NmrA-like protein was reported in humans, designated as HSCARG, while, in animals such as mice, rats, and bovines, it was known as NMRAL1 [17]. NMRAL1 negatively regulates innate immunity by suppressing the activities of the NF-κB and RIG-I-like receptor (RLR) pathway [18]. It serves as a crucial link connecting the cellular redox status with other signaling pathways. It inhibits NO production through interacting with argininosuccinate synthetase (AS) [18]. While the regulation of NO by NMRA-like proteins is well-established in other contexts, their specific roles in TB infection remain unexplored.

Previously, we noticed another unannotated gene ENSBTAG00000011305, which contains a NmrA domain beside the *NMRAL1* gene (ENSBTAG00000010015), and designated it as the *NMRAL2_Bovine* (hereafter *NMRAL2*). This study aimed to demonstrate the function of the NMRAL2 in the host defense against *M. bovis* infection related to the NO production and inflammatory response. As a result, *NMRAL2* knockout and knockdown led to a marked increase in NO production, and a reduction in cellular death induced by *M. bovis* and number of intracellular *M. bovis*. These findings contribute to a more comprehensive understanding of TB pathogenesis and the development of novel control measures for both human and animal TB.

## 2. Materials and Methods

### 2.1. Cell and Bacterial Culture

The embryonic bovine lung (EBL) cell lines were generously provided by M. Heller from the Friedrich-Loeffler-Institute in Jena, Germany. These cells were maintained in Dulbecco’s Modified Eagle’s Medium (DMEM; Gibco, Melbourne, Australia), which was supplemented with non-essential amino acids (NEAA, Gibco, Melbourne, Australia) and 10% heat-inactivated fetal bovine serum (FBS, Gibco, Melbourne, Australia). The HEK293FT cell line was procured from the Cell Bank of the Chinese Academy of Sciences in Shanghai, China, and cultured in DMEM supplemented with 10% FBS, 100 U/mL penicillin, and 100 µg/mL streptomycin, under conditions of 37 °C and 5% CO_2_.

*M. bovis* was obtained from Professor Junyan Liu from Wuhan University. Professor Luiz Bermudez from Oregon State University supplied BCG-Pasteur strain. BCG-Pasteur strain expressing red fluorescent protein DsRed (BCG-red) was provided by Professor Gang Cao from Huazhong Agriculture University. Bacterial strains were cultured in Middlebrook 7H9 broth with 0.5% glycerol, 10% oleic acid-albumin-dextrosecatalase (OADC; BD PharMingen, San Diego, CA, USA), and 0.05% Tween 80, or on Middlebrook 7H11 agar plates containing 0.5% glycerol and 10% OADC.

Prior to infection, the optical densities of bacterial cultures were standardized at 600 nm (OD_600_) to achieve the desired multiplicity of infection (MOI). The cultures underwent centrifugation at 5000× *g* for 10 min, after which the bacterial pellet was resuspended in a medium and dispersed using an insulin syringe. Subsequently, a 100 μL aliquot of the 10-fold serially diluted bacterial suspension was plated on Middlebrook 7H11 agar to quantify viable bacteria as colony-forming units (CFUs).

### 2.2. Construction of Gene Knockout Cell Line

To generate the *NMRAL2* knockout cell line (NMRAL2-KO), *NMRAL2* sgRNA sequence (NMRAL2_sgRNA-F: CACCGGTATCTCGGTTGCTGATAT; NMRAL2_sgRNA-R: AAACATATCAGCAACCGAGATACC) were inserted into the lentiCRISPR-V2 vector. This construct was subsequently co-transfected with the pMD2.G and psPAX2 plasmids into HEK293FT cells using JetPRIME reagent (Polyplus, Illkirch, France), following the manufacturer’s protocol. After 60 h post-transfection, the supernatants of the cell cultures were collected, filtered using 0.45 μm low protein binding membrane (Millipore Corporation, Billerica, MA, USA), and centrifuged at 150,000× *g* for 2.5 h at 4 °C. Lentivirus pellets with *NMRAL2* sgRNA were resuspended in PBS (pH 7.4), aliquoted, and stored at −80°C. Following infection with the lentivirus, EBL cells underwent selection for successful infection using puromycin (Invivo Gen, San Diego, CA, USA) at a concentration of 5 μg/mL. The knockout of the *NMRAL2 gene* was confirmed using Sanger sequencing and RT-qPCR analysis. The specific forward primers (5ʹ-AGCGACTCAGTGTTACATGGTG-3ʹ) and reverse primers (5ʹ-TTCACCCCAACTCTGCTGTG-3ʹ) were utilized for polymerase chain reaction (PCR) amplification, followed by Sanger sequencing (Tsingke, Beijing, China).

### 2.3. RNAi

siRNA of *NMRAL2* and *NMRAL1_Bovine* (Si-*NMRAL1*: CAGUGCAUUUCGGUCAGACCUGAUU; Si-*NMRAL2*: UGGCUUCUGCUAAGCUACCUCUGAG) gene was synthesized by Tsingke (Tsingke, Beijing, China). EBL cells were cultured in 12-well plates and transfected with 80 pmol of siRNA utilizing Lipofectamine 2000 (Invitrogen, Carlsbad, CA, USA) when they reached approximately 60% confluence, in accordance with the manufacturer’s instructions. A negative control siRNA (NC siRNA) was employed as a control. Subsequent to a 48 h post-transfection incubation period, the cells were subjected to further functional analyses.

### 2.4. RNA-Seq

Total RNA extraction was performed utilizing the Trizol Reagent (Invitrogen, Carlsbad, CA, USA). The concentration, quality, and integrity of the extracted RNA were evaluated using a NanoDrop spectrophotometer (Thermo Scientific, Waltham, MA, USA). Subsequent sequencing libraries were prepared following established protocols. mRNA was selectively isolated from the total RNA using poly-T oligonucleotide-attached magnetic beads. Fragmentation of the mRNA was achieved through the application of divalent cations at elevated temperatures within an Illumina proprietary fragmentation buffer. The synthesis of first-strand complementary DNA (cDNA) was performed utilizing random oligonucleotides in conjunction with SuperScript II reverse transcriptase. This was followed by the synthesis of the second-strand cDNA, which was achieved using DNA Polymerase I and RNase H. To convert any remaining overhangs into blunt ends, exonuclease and polymerase activities were employed, after which the enzymes were removed. Subsequently, Illumina paired-end (PE) adapted oligonucleotides were ligated to the DNA fragments, following adenylation of their 3′ ends, to facilitate hybridization. To preferentially select cDNA fragments ranging from 400 to 500 base pairs in length, library fragments were purified utilizing the AMPure XP system (Beckman Coulter, Beverly, CA, USA). DNA fragments with adaptor molecules ligated at both ends were selectively enriched through a 15-cycle PCR reaction employing the Illumina PCR Primer Cocktail. The resulting products were then purified again and quantified. The sequencing library was subsequently processed using the NovaSeq 6000 platform (Illumina, San Diego, CA, USA) by Shanghai Personal Biotechnology Co., Ltd (Shanghai, China). The RNA sequencing services were facilitated by Personal Biotechnology Co., Ltd., located in Shanghai, China. Data analysis was conducted utilizing the online platform Personalbio GenesCloud (https://www.genescloud.cn, accessed on 11 August 2022). Furthermore, genes associated with the inflammatory response were randomly selected for validation. The primer sequences used for RT-qPCR are detailed in Table 1.

### 2.5. RT-qPCR

Total RNA was extracted from the cells utilizing the TRIzol Reagent (Invitrogen, Carlsbad, CA, USA). cDNA synthesis was performed by reverse transcription of the isolated RNA using the HiScript III RT SuperMix with gDNA Eraser (Vazyme, Nanjing, China) in a reaction volume of 20 μL. Amplification of target genes was achieved using a reaction mixture comprising 5 μL of SYBR Green Mix, 0.5 μL of gene-specific primers, and 3 μL of double-distilled H_2_O, culminating in a total volume of 10 μL. The PCR protocol was conducted using a Bio-Rad IQ5 instrument (Bio-Rad, Hercules, CA, USA) and comprised an initial denaturation phase of 5 min at 95 °C, followed by 40 cycles consisting of 10 s at 95 °C and 30 s at 60 °C.

### 2.6. EdU Fluorescence and CCK-8 Assays

Cell viability and proliferation were assessed utilizing the CCK-8 and EdU fluorescence assays. NMRAL2-KO and WT EBL cells were initially seeded into 96-well plates. Each well was subsequently treated with CCK-8 solution (Dojindo, Kumamoto, Japan), and absorbance was recorded at 450 nm. For the EdU fluorescence assay, NMRAL2-KO and WT EBL cells were cultured in 12-well plates. The EdU cell proliferation assay was then performed using the BeyoClick EdU kit (Beyotime Biotechnology, Shanghai, China) with Alexa Fluor 488, following the manufacturer’s instructions. Fluorescence microscopy was used to visualize treated cells, and ImageJ software (ImageJ software, 1.52a; National Institutes of Health, Bethesda, MD, USA) calculated the proportion of EdU-positive cells. Images were taken from a random field of view in each of three independent wells.

### 2.7. Western Blotting and Antibodies

Cells were lysed at 4 °C for 30 min using a cell lysis buffer supplemented with a 1× complete protease inhibitor cocktail (Roche, Mannheim, Germany). Cellular debris was eliminated by centrifugation at 12,000× *g* for 15 min at 4 °C. The supernatant derived from the lysed cells was subsequently subjected to heating at 95 °C for a duration of 5 min in the presence of loading buffer. Proteins were then denatured, resolved via SDS-PAGE, and transferred onto a polyvinylidene fluoride (PVDF; Millipore Corporation, Bedford, MA, USA) membrane. The PVDF membranes underwent a blocking procedure for a minimum duration of three hours utilizing Tris-buffered saline with Tween-20 (TBST) supplemented with 5% skim milk. Subsequently, the membranes were incubated with primary antibodies in TBST, a process conducted either overnight at 4 °C. For Western blot analysis, primary antibodies from Cell Signaling Technology (Cell Signaling Technology, Boston，MA, USA) were utilized, including rabbit monoclonal antibodies for phospho-p65 (Ser536) (93H1) (#3033), p38 (#9212), phospho-p38 (Thr180/Tyr182) (#9211), p44/42 (Erk1/2) (137F5) (#4695), phospho-p44/42 (Erk1/2) (Thr202/Tyr204) (D13.14.4E) XP^®^ (#4370), JNK (56G8) (#9258), and phospho-SAPK/JNK (Thr183/Tyr185) (98F2) (#4671). Mouse monoclonal antibodies for NF-κB subunit p65 (L8F6) (#6956) was also obtained from the same supplier. For loading controls, Anti-β-actin antibody was obtained from Proteintech (Proteintech, Wuhan, China). Secondary antibodies specific to mouse and rabbit (Abbkine, Wuhan, China) were utilized in TBST for a duration of one hour at room temperature. Protein detection was subsequently performed using ECL Prime Western blot detection reagents (Bio-Rad, Hercules, CA, USA).

### 2.8. Invasion and Adhesion Assays

In the invasion and adhesion assay, both NMRAL2-KO and wild-type (WT) EBL cells were exposed to *M. bovis* at an MOI of 10:1. For the invasion assay, cells were incubated at 37 °C for 1 h, whereas, for the adhesion assay, incubation was conducted at 4 °C for 30 min. Following incubation, the cells underwent three washes with 1× phosphate-buffered saline (PBS; Gibco, Melbourne, Australia) and were subsequently lysed using 0.025% Tween-20 for a duration of 10 min. The resulting cell lysate was subjected to a 10-fold serial dilution prior to being plated on 7H11 agar. Bacterial colony-forming units (CFUs) were quantified after an incubation period of three weeks.

### 2.9. Intracellular Survival Assay

NMRAL2-KO and WT EBL cells were inoculated with *M. bovis* at an MOI of 10. Following infection, the cells underwent three washes with PBS and were subsequently incubated in fresh DMEM supplemented with 100 μg/mL gentamicin to eradicate extracellular bacteria. The cells were then washed and lysed using 0.025% Tween-20 for 10 min at intervals of 0, 24, 48, and 72 h post-infection. The resulting lysates were subjected to serial ten-fold dilutions and plated on 7H11 agar. Colony formation was assessed after an incubation period of 3 to 4 weeks at 37 °C.

### 2.10. Analysis of Intracellular NO Production

The release of intracellular NO was evaluated utilizing a fluorescent microscope in conjunction with the NO-sensitive fluorescence probe DAF-FM DA (Beyotime Biotechnology, Shanghai, China). Both NMRAL2-KO and WT cells were exposed to *M. bovis* for a duration of 24 h, followed by treatment with 1 μM DAF-FM DA for 20 min. Post-treatment, the medium was aspirated, and the cells underwent three washes with phosphate-buffered saline (PBS). Confocal microscopy (Nikon, Tokyo, Japan) was employed to capture fluorescence images, which were subsequently analyzed to determine cellular fluorescence intensity. Additionally, quantitative measurement of NO concentration was performed using the Griess reagent kit assay (Beyotime Biotechnology, Shanghai, China). The concentration of NO in cell culture supernatants was quantified utilizing a Griess-reaction-based NO assay kit. Both NMRAL2-KO and WT cells were infected with *M. bovis* for durations of 0, 24, and 48 h, adhering to the manufacturer’s protocol for sample preparation.

### 2.11. Statistical Analysis

All assays and experiments were conducted in triplicate, with data reported as means ± standard errors of the mean (SEMs) derived from these triplicates. Statistical analyses were performed utilizing GraphPad Prism software (GraphPad Prism, version 9.0, Boston, MA, USA). The Student’s *t*-test was applied for comparisons between two groups, whereas analysis of variance (ANOVA) was utilized for comparisons involving more than two groups. Statistical significance was categorized as non-significant (ns, *p* > 0.05), * p < 0.05, ** p < 0.01, and *** p < 0.001.

## 3. Results

### 3.1. ENSBTAG00000011305 Gene Belongs to the NmrA Protein Family

**Figure 1:**
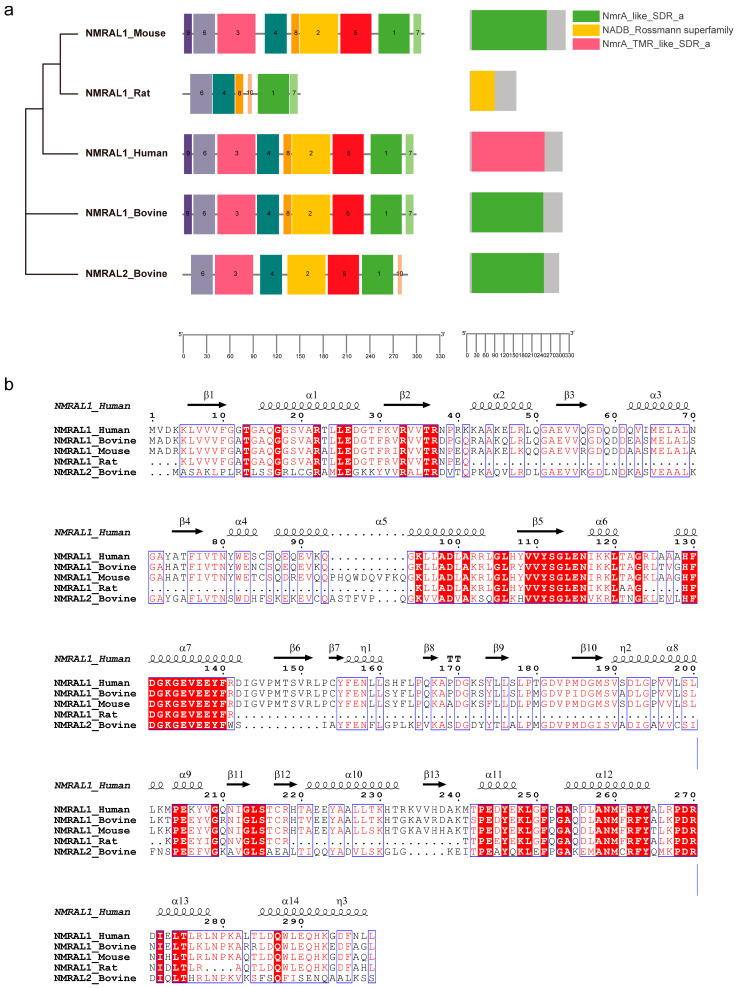
Bioinformatic analysis on domain and amino acid similarity of NMRAL2 with other NmrA_like proteins. (**a**) The schematic illustration depicts the NmrA-like domain and its conserved motifs within the NMRAL2 protein and its homologs. The analysis of conserved domains in NMRAL2 and its homologs was performed using the NCBI Conserved Domain Database (CDD) search tool. Additionally, the identification of conserved motifs within these protein sequences was carried out using the MEME Suite motif search tool. (**b**) A multiple sequence alignment of protein homologs related to NMRAL2 was performed using Clustal Omega, and the results were visualized with ESPript 3.0. The red boxes represent identity, and the blue boxes indicate partial similarity.

To characterize the NmrA-like protein family across species, we conducted a domain analysis using the MEME Suite motif search tool. We compared two bovine genes of the NmrA-like proteins NMRAL1_Bovine (ENSBTAG00000010015) and NMRAL2 (ENSBTAG00000011305) with human, mouse, and rat species and identified highly similar conserved motifs within these genes in these species sharing motifs 1, 4, 6, 7, and 8. These suggest that these motifs may serve as core elements of the NmrA-like family (Figure 1a). Additionally, we discovered unique motif 10 only exists in both NMRAL1_Rat and NMRAL2. To further explore the conserved domains within the NmrA-like family genes, we utilized Batch CD-Search and revealed highly similar domains, including the NADB_Rossmann Superfamily (cl21454), NmrA_TMR_like_SDR_a (cd08947), and NmrA_like_SDR_a (cd05251). Notably, both NmrA_TMR_like_SDR_a and NmrA_like_SDR_a are part of the NADB_Rossmann Superfamily. In conclusion, compared to the NMRAL1 , NMRAL2 shared motif 1–6, but has a unique motif 10, and lacks motif 7–9.

In addition, to investigate the conservation of protein sequences among NmrA-like family members, the similarity at the amino acid level was analyzed. We conducted multiple sequence alignments of human, mouse, rat, and bovine protein sequences. The analysis revealed conserved regions in the middle and C-terminal portions of NmrA-like protein sequences, consistent with the domain analysis results. This suggests that the conserved amino-terminal sequence serves as the primary sequence basis for the NmrA-like family domain (Figure 1b). In comparison to bovine NMRAL1, the bovine NMRAL2 protein demonstrates a deletion of 11 amino acids at specific functional sites, with a sequence similarity of 47.6%. Conversely, the sequence similarity between bovine NMRAL1 and human NMRAL1 is 86.0%. These observations indicate potential functional divergences in NMRAL2 relative to NMRAL1, meriting further investigation to elucidate its distinct role and significance.

### 3.2. NMRAL2 Enhances Intracellular Survival of M. bovis and Cell Death

**Figure 2:**
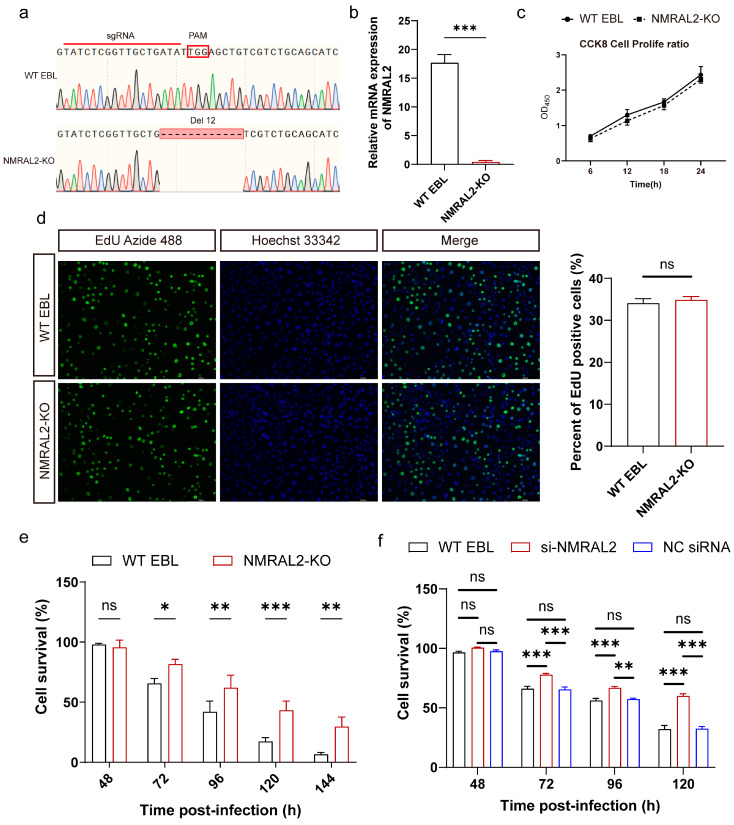
The effect of NMRAL2 on cell survival during *M. bovis* infection determined with CCK8 assay. (**a**) DNA sequence analysis verified the mutation in clonal *NMRAL2* KO cells. In the KO cells, deleted bases are highlighted in red, while PAM sites are enclosed in a red box. (**b**) RT-qPCR analysis confirmed deficiency of *NMRAL2* expression in knockout cell lines. (**c**,**d**) The impact of *NMRAL2* on EBL cell proliferation was assessed through the CCK-8 assay and the EdU assay. The blue fluorescence represents the nucleus stained by DAPI and the green fluorescence represents EdU-positive cells. (**e**,**f**) The impact of *NMRAL2* knockout and knock-down on cell viability following infection with *M. bovis*. Cell survival of WT, NMRAL2-KO, and si-NMRAL2 EBL cells post *M. bovis* infection was evaluated using the CCK8 assay. A two-tailed unpaired *t*-test and a two-way ANOVA were employed to evaluate the statistical significance between groups, utilizing data from three independent replicates. Statistical significance is denoted as follows: *p < 0.05, ** p < 0.01, *** p < 0.001, and ns for no significant difference.

To investigate the function of NMRAL2, we utilized the CRISPR/Cas9 system to knock out the *NMRAL2* gene in the EBL cell line. Single-clone screening followed by Sanger sequencing confirmed a 12 bp deletion in the *NMRAL2* gene in the single clone cell line (Figure 2a). An RT-qPCR analysis further verified the successful knockout, and this cell line was designated as NMRAL2-KO (Figure 2b). Furthermore, the impact of the *NMRAL2* knockout on cellular proliferation was assessed utilizing EdU staining and CCK8 assays. The findings demonstrated that the deletion of the *NMRAL2* gene had no impact on the proliferation of EBL cells (Figure 2c,d). To investigate the function of NMRAL2 in the context of *M. bovis* infection, both NMRAL2-KO and WT cells were infected with *M. bovis* at an MOI of 100. The results from CCK8 assays revealed that the knockout of *NMRAL2* significantly attenuated *M. bovis*-induced cell death (Figure 2e).

Furthermore, we examined the impact of NMRAL1 and NMRAL2 on *M. bovis* infection through targeted siRNA-mediated knockdown. The RT-qPCR analysis revealed that the knockdown efficiencies for *NMRAL1* and *NMRAL2* were 60% and 55%, respectively (Figure S1a). Notably, *NMRAL2* siRNA knockdown resulted in a 24% enhancement in cell survival (Figure 2f). In contrast, the knockdown of *NMRAL1* using siRNA did not lead to a statistically significant enhancement in cell survival; rather, it induced a degree of cell death (Figure S1b).

**Figure 3:**
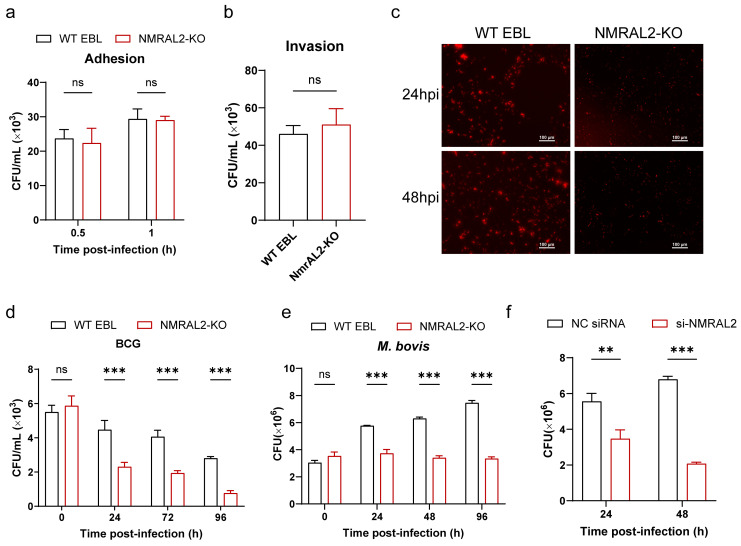
The impact of *NMRAL2* knockout or knockdown on intracellular *M. bovis* viability. (**a**,**b**) Assessing the impact of NMRAL2 on *M. bovis* invasion and adhesion capabilities. EBL cells, with or without *NMRAL2* expression, were infected with *M. bovis* at an MOI of 10. The invasion assay was conducted at 37 °C for 1 h, while the adhesion assay was performed at 4 °C for 30 min. The CFU assay quantified *M. bovis* numbers. (**c**–**f)** The impact of *NMRAL2* knockout or knockdown on intracellular *M. bovis* survival. WT, NMRAL2-KO, and si-NMRAL2 EBL cells were infected with *M. bovis* or BCG. Intracellular survival of *M. bovis* or BCG was evaluated using DsRed-expressing BCG (**c**) and plate counting methods (**d**–**f**). A two-tailed unpaired *t*-test and a two-way ANOVA were employed to assess the statistical significance of differences among various groups, utilizing three independent replicates for each analysis. ** p < 0.01 and *** p < 0.001 indicate statistically significant differences, and ns for no significant difference.

To determine whether NMRAL2 affects the adhesion and invasion of *M. bovis*, we conducted adhesion experiments at an MOI of 10 at 4 °C for 0.5 h and 1 h. The results demonstrated that *NMRAL2* knockout did not affect the adhesion of *M. bovis* to EBL cells (Figure 3a). Similarly, under conditions of MOI 10 at 37 °C for 0.5 h, there was no impact on *M. bovis* invasion in *NMRAL2* knockout and WT cells (Figure 3b).

Next, we examined whether the improved host cell survival was linked to the control of intracellular mycobacterial growth in NMRAL2-KO cell lines or not; we infected NMRAL2-KO and WT cells with *M. bovis* and BCG at a MOI of 10. The results showed that *NMRAL2* knockout decreased the intracellular survival of both *M. bovis* and BCG by 44% and 27% (*p* < 0.001) (Figure 3c–e). Accordingly, during post-infection with *M. bovis*, we observed that the knockdown of *NMRAL2* reduced the intracellular survival of *M. bovis* by 30.4%, less than that in NMRAL2-KO cells (Figure 3e,f).

These findings indicate that NMRAL2 facilitates the intracellular survival of *M. bovis* without influencing its adhesion to or invasion of EBL cells.

### 3.3. NMRAL2 Plays Significant Roles in Suppressing Host Innate Immune Responses

**Figure 4:**
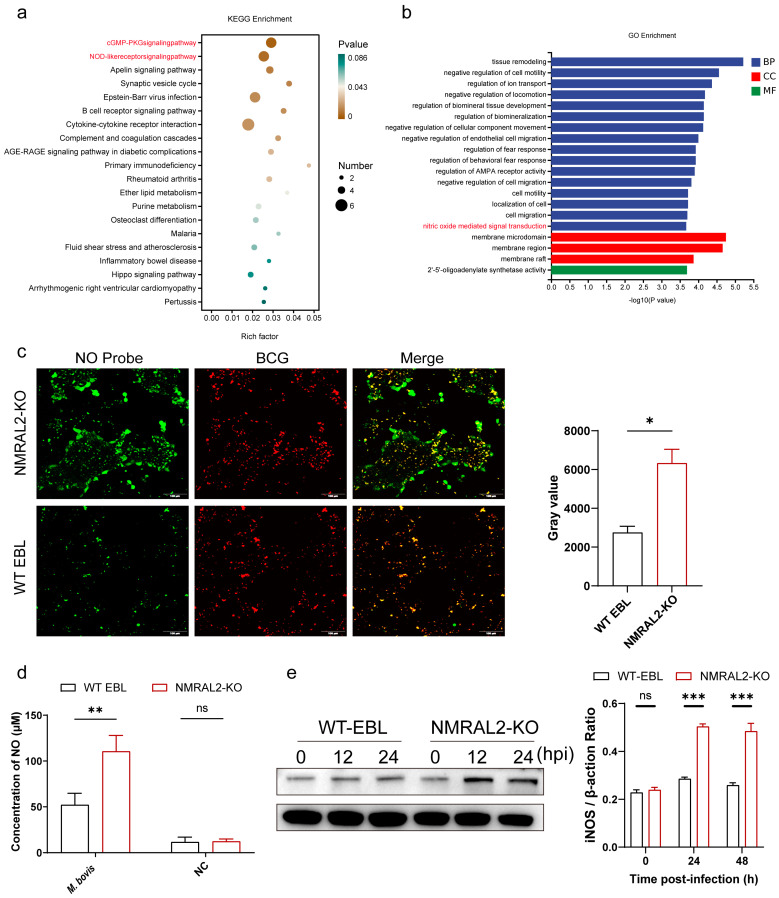
Impact of *NMRAL2* knockout on host gene expression during *M. bovis* infection. (**a**,**b**) Gene Ontology (GO) and Kyoto Encyclopedia of Genes and Genomes (KEGG) analysis for differentially expressed genes from the RNA-seq data comparing NMRAL2-KO-*M. bovis* versus WT-*M. bovis*. BP: biological process, MF: molecular function, and CC: cellular component. Words marked in red indicate the representative pathways that the research focuses on. (**c**) Quantification of NO concentrations. Intracellular NO level detected using the NO probe DAF-FM DA (Green). EBL cells were uninfected and infected with the indicated BCG (DsRed) at a MOI of 10 for 6 h. Cells were incubated with 1μM DAF-FM DA for an additional 30 min before being analyzed via confocal microscopy observation (Left). Scale bars: 100 μm. Ten random images were captured from each sample, and fluorescence intensities were quantified using ImageJ software. (**d**) Quantitative analysis of intracellular NO levels by Griess Reagent. (**e**) Immunoblotting analysis of iNOS expression in WT and NMRAL2-KO EBL cells. Various EBL cells were infected by *M. bovis* at an MOI of 10 for 48 h, followed by immunoblotting. The statistical significance among the different groups was evaluated utilizing a two-tailed unpaired *t*-test and a two-way ANOVA, based on three independent replicates. * p < 0.05, ** *p* < 0.01, and *** p < 0.001 indicate statistically significant differences, and ns for no significant difference.

To explore how the *NMRAL2* gene affect the intracellular survival of *M. bovis*, we performed transcriptome sequencing on NMRAL2-KO and WT EBL cells infected with *M. bovis*. As a result, we identified 24 upregulated and 97 downregulated genes in NMRAL2-KO cells compared to WT EBL cells. Further analysis demonstrated that these differentially expressed genes (DEGs) were enriched in pathways such as NO-mediated, cGMP-PKG, NOD-like receptor, and cytokine–cytokine receptor interaction signal transduction pathways. Notably, among them, several pathways that negatively regulate host immune responses were also enriched (Figure 4a,b).

The enrichment of NO-mediated signal transduction is particularly of interest (Figure 4b). NMRAL2 contains the NmrA functional domain, which is known to regulate NO production. To assess the NO response in EBL cells, we used an NO probe to assess the NO response in EBL cells at 48 h post-infection with BCG. The results indicated a significant increase in NO concentrations in *NMRAL2* knockout cells, as evidenced by the enhanced green fluorescence intensity (Figure 4c). A quantitative analysis using the Griess Reagent confirmed that NO levels in *NMRAL2* knockout cells were significantly higher than in WT EBL cells 48 h after *M. bovis* infection (*p* < 0.05), while no difference was observed between the uninfected groups (Figure 4d). Since intracellular NO is produced by nitric oxide synthase (NOS), we further examined the activation of inducible NOS (iNOS) following *M. bovis* infection and determined that iNOS expression was significantly induced in *NMRAL2* KO cells at both 24 and 48 h post-infection (Figure 4e).

In conclusion, these findings confirm the reduced intracellular survival of *M. bovis* and increased NO production in *NMRAL2* KO cells.

### 3.4. NMRAL2 Gene Knockout Affects Activation of Innate Immune Response Induced by M. bovis Infection

**Figure 5:**
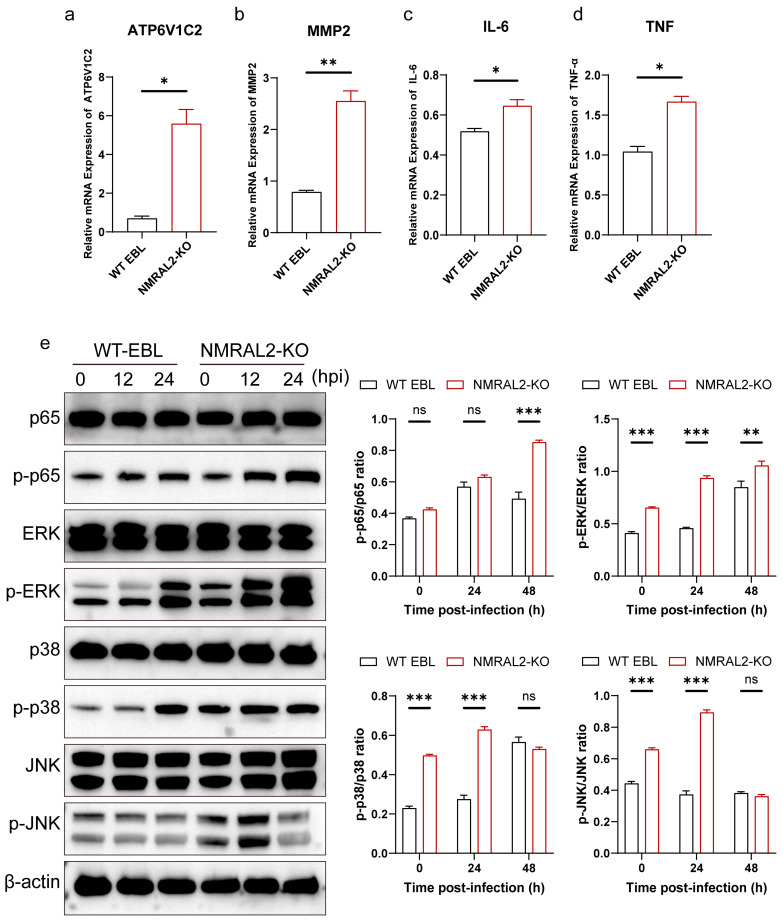
The effect of NMRAL2 on cell inflammation during *M. bovis* infection. (**a**–**d**) Expression of proinflammatory cytokines in EBL cells. EBL cells, both WT and NMRAL2-KO, were infected with *M. bovis* for 24 h. The mRNA levels of ATP6V1C2 (**a**), MMP2 (**b**), IL-6 (**c**), and TNF-α (**d**) were assessed via RT-qPCR. The mRNA levels of these cytokines were normalized to those of β-actin mRNA. (**e**) The knockout of *NMRAL2* resulted in an enhanced activation of the NF-κB and MAPK signaling pathways in response to *M. bovis*; EBL cells were exposed to *M. bovis* at an MOI of 10 for a designated infection period. Subsequently, the cells were lysed on ice for 30 min and analyzed via Western blot to determine the total and phosphorylated levels of the proteins p65, p38, JNK, and ERK1/2. The grayscale values of the phosphorylated to total protein ratios (p-p65/p65, p-p38/p38, p-JNK/JNK, and p-ERK/ERK) were normalized against β-actin. The statistical significance among the different groups was evaluated utilizing a two-tailed unpaired *t*-test and a two-way ANOVA, based on three independent replicates. Significance levels are denoted as follows: * *p* < 0.05, ** *p* < 0.01, and *** *p* < 0.001, and ns for no significant difference.

The NmrA functional domain in NMRAL2 is implicated in the regulation of the host NF-κB signaling pathway, in addition to its role in the NO signaling pathway. The transcriptome sequencing results confirmed that *NMRAL2* knockout can activate multiple innate immune pathways including host inflammatory responses (Figure 4a,b). We selected four genes regulated by the NF-κB signaling pathway involved in *M. bovis* infection for further validation. Using RT-qPCR, we evaluated the differential expression of genes including *IL-6*, *TNF-α*, *ATP6V1C2*, and *MMP2*. In NMRAL2-KO cells, *ATP6V1C2* and *MMP2* gene expression was significantly upregulated (*p* < 0.05), aligning with our transcriptome sequencing results (Figure 5a,b). Additionally, at 48 h after *M. bovis* infection, the KO cell line showed a significant increase in inflammatory cytokines IL-6 and TNF-α (*p* < 0.05) (Figure 5c,d). These findings indicate that NMRAL2 acted as a negative immune regulator during *M. bovis* infection.

We further examined the changes in signaling molecules within the classical NF-κB and MAPK pathways in NMRAL2-KO and WT cells following *M. bovis* infection. At 48 h post-infection, we observed a significant increase in the phosphorylation levels of p65 in the NF-κB pathway, and p38, ERK, and JNK in the MAPK pathway significantly increased, with the ERK pathway activation being the most pronounced (Figure 5e). Overall, *NMRAL2* knockout led to the activation of both the NF-κB and MAPK pathways and promoted the expression of key inflammatory cytokines during *M. bovis* infection. This demonstrates that NMRAL2 is involved in negatively regulating the host immune response, suppressing the inflammatory response during *M. bovis* infection.

## 4. Discussion

In this study, we elucidate the pivotal role of NMRAL2 in modulating the response of EBL cells to *M. bovis* infection. Our results demonstrate that the knockout of *NMRAL2* significantly reduces cell death induced by *M. bovis*, revealing a previously unrecognized mechanism that enhances host cell survival against *M. bovis* infection. This discovery underscores the importance of NMRAL2 in NO production, a key factor in activating anti-tuberculosis innate immune pathways. The regulation of NO synthesis and the immune response by NMRAL2 provides novel insights into the dynamics of host–pathogen interactions. Consequently, this study offers a new perspective regarding the interplay between cellular defense mechanisms and *M. bovis* infection.

The modulation of NO signaling involves not only redox molecule dynamics but also interactions with regulatory proteins like NMRAL1 [18]. NMRAL1 directly affects NO synthesis by reducing argininosuccinate synthetase activity [18]. Our findings align with this, as the presence of specific conserved motifs in NMRAL2 indicates potential functional similarities with NMRAL1. However, the distinct outcomes observed when knocking down *NMRAL1* versus *NMRAL2* highlight the functional divergence within this protein family. The broader NmrA protein family, including PadA, QOR2, and PcNMRAL1, is known for binding to coenzymes NAD(H) or NADP(H) without dehydrogenase activity [17], characterized by a short-chain dehydrogenase/reductase-like structure. This family features an N-terminal Rossmann fold essential for coenzyme binding [19]. Despite the topological resemblance to NmrA, NMRAL1 shows a distinct affinity for reduced NADPH due to structural variations, such as the absence of NmrA-like Thr-276 in its C-terminal region [17]. We also found that the 276th amino acid of NMRAL2 is His, while the 276th amino acid of NMRAL1 is Leu. Such variations underscore the functional divergence within the NmrA protein family, particularly in their respective roles in NO regulation and redox sensing. Our results suggest that minor structural changes can lead to functional differences in the NmrA protein family.

Our comparative analyses across species highlight the high conservation of NMRAL1, suggesting its ubiquitous role in biological functions, particularly in NO production and cellular survival mechanisms. The structural similarity of NMRAL2 to bovine-derived NMRAL11 implies a potential functional overlap. However, our results show that knocking down both *NMRAL1* and *NMRAL2* in EBL cells yields distinct outcomes. Specifically, *NMRAL1* knockdown led to more cell death compared to both *NMRAL2* knockdown and WT cells. Conversely, knocking out or knocking down *NMRAL2* enhanced cell survival following *M. bovis* infection. This discrepancy may stem from structural differences between NMRAL1 and NMRAL2. Notably, while NMRAL2 shares a structure and motifs with NMRAL1, it lacks Motifs 7–9 and has an additional Motif 10 at its C-terminus. The absence of certain motifs in NMRAL2_Bovine, combined with an additional motif at its C-terminus, may account for the functional distinctions between the two homologs proteins, NMRAL2 and NMRAL1.

NMRAL1 is known to regulate NO production by sensing cellular redox states inhibiting excessive oxidative stress through a negative feedback mechanism, preventing cellular damage [20]. NMRAL1 is an important regulator of NO playing a dual role, and its absence may disrupt the cellular redox balance, leading to cytotoxicity, with excessive levels causing cellular damage [21,22,23]. Therefore, we hypothesized that the knockout of *NMRAL2* may lead to a moderate increase in NO levels, effectively controlling infection without inducing excessive inflammatory damage. Additionally, NMRAL2 may function as an auxiliary pathway for NO regulation, potentially increasing NO production upon deletion, thereby enhancing *M. bovis* clearance and promoting cell survival. However, these hypotheses warrant further investigation to elucidate the specific molecular interactions and pathway analyses. Furthermore, the evolutionary aspects of the NmrA-like protein family, particularly the differences and similarities between species, remain an open area for exploration. Gene family analyses indicates that *NMRAL2* may have evolved through duplication and mutation events within the *NMRAL1* gene family, a hypothesis that requires validation through evolutionary biology studies.

NMRAL1 acts a negative regulator of innate immune responses, inhibiting cytokine-induced NF-κB activation [24,25,26]. In resting cells, NF-κB is sequestered in the cytoplasm by IκB proteins, which suppress the transcriptional activity of the p50 and p65 subunits [27]. In the classical NF-κB signaling pathway, cytokines like TNF-α or IL-1β trigger the ubiquitination and degradation of IκB, allowing p50 and p65 to translocate to the nucleus and regulate transcription [28,29]. NMRAL1 interacts with IKKβ and NEMO, influencing post-translational modifications such as IKKβ dephosphorylation by PP2A and NEMO deubiquitination by USP7 [24,25,26]. Given the structural similarities between NMRAL2 and NMRAL1, it is plausible that NMRAL2 exert similar functions within the NF-κB pathway. Our research indicates that the knockout of *NMRAL2* leads to the significant activation of innate immune pathways, marked by an increased phosphorylation of the p65 signaling molecule, suggesting its involvement in the NF-κB signaling cascade. However, whether NMRAL2 modulates this pathway through the same strategies requires further investigation to fully elucidate its role in innate immune response regulation.

Our research on bovine NMRAL2 offers insights relevant to human TB, due to the shared pathogenic mechanisms between *M. bovis* and *M. tb*. Although these mycobacterial strains differ in host specificity, they utilize analogous strategies to circumvent host immune responses [30,31], indicating conserved roles in pathogenesis. The NmrA-like protein family, encompassing NMRAL1 and NMRAL2, is highly conserved across species and plays crucial roles in immune modulation. In humans, NMRAL1 (HSCARG), a homolog of NMRAL2, has been shown to modulate immune responses, particularly by influencing NO production and cytokine expression [17]. In this study, we observed that the knockdown of *NMRAL2* in bovine cells facilitated the clearance of *M. bovis* and enhanced host cell survival by augmenting NO levels and pro-inflammatory cytokine production. Conversely, the knockdown of *NMRAL1* in bovine cells resulted in increased cell death. These findings highlight the functional diversity within the NmrA protein family and suggest a conserved role for NMRAL1 in the pathogenesis of TB. We hypothesize that the moderate inhibition of NMRAL1 in humans could enhance NO levels, thereby facilitating pathogen clearance while minimizing cytotoxicity and tissue damage. Further research is required to confirm the role of NMRAL1 in human TB, which may uncover novel strategies for host-directed therapies targeting redox-sensitive pathways.

## 5. Conclusions

**Figure 6:**
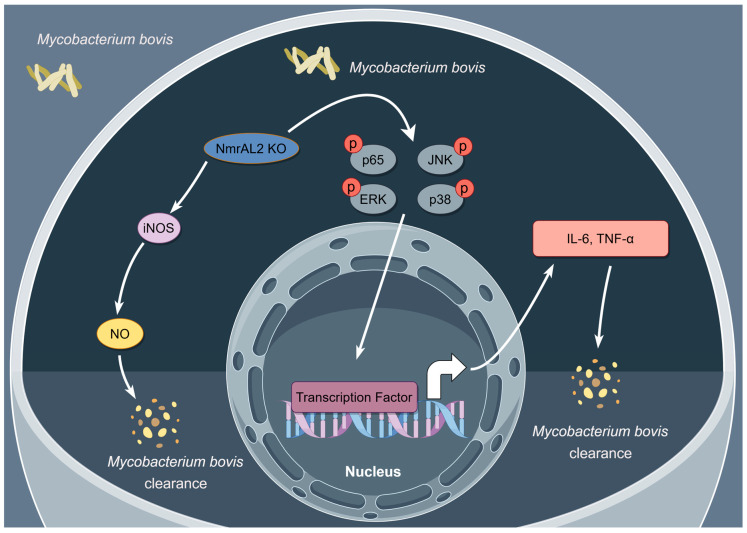
Proposed model of NMRAL2 function during *M. bovis* infection. This schematic illustrates the proposed role of NMRAL2 in bovine lung epithelial cells during *M. bovis* infection, focusing on the effects observed following *NMRAL2* knockout. The absence of NMRAL2 results in reduced intracellular survival of *M. bovis*, along with enhanced production of NO and increased expression of proinflammatory cytokines. The upregulation of these cytokines is mediated through activation of the NF-κB and MAPK signaling pathways, highlighting NMRAL2 as a negative regulator of the host immune response to *M. bovis*.

In conclusion, our study firstly demonstrated the significant role of NMRAL2 in regulating NO production and proinflammatory cytokines responding to *M. bovis* infection and, thereby, influencing the survival of infected cells and the intracellular survival of *M. bovis* (Figure 6). Therefore, the findings underscore the critical role of NMRAL2 in the host defense against *M. bovis* infection, providing valuable insights into the pathogenesis of human and bovine tuberculosis and potential therapeutic targets for host-directed therapies.

## Figures and Tables

**Table 1:** Primers utilized in this research.

Primer Name	Sequence (5′–3′)
*bNMRAL1*-F	ACAAAGAGGACTTCGCAGGG
*bNMRAL1*-R	TGGAAGCCATCTGTGAGAACA
*bNMRAL2*-F	TTTGGGGAAAGAAATCACCCCAG
*bNMRAL2*-R	CGGTGGGTGAGCTGAATGTC
*bMMP2*-F	ATCGTCTTCGACGGCATCTC
*bMMP2*-R	TTCGCCAGATGAATCGGTCC
*bATP6V1C2*-F	CAGAATGTCCACGACGCTCA
*bATP6V1C2*-R	ATGCGTCTGTCTCTTGCAGT
*bIL-6*-F	ACCCCAGGCAGACTACTTCT
*bIL-6*-R	CCCAGATTGGAAGCATCCGT
*bTNF-α*-F	CTCCATCAACAGCCCTCTGG
*bTNF-α*-R	GAGGGCATTGGCATACGAGT
*β-actin*-F	AGCAAGCAGGAGTACGATGAG
*β-actin*-R	ATCCAACCGACTGCTGTCA

## Data Availability

The datasets generated in this Articles/Supplementary Materials are available upon request from the corresponding authors. The original data presented in the study are openly available in the SRA database at https://www.ncbi.nlm.nih.gov/bioproject/PRJNA1178837/, accessed on 31 October 2024.

## Supplementary Materials

The following supporting information can be downloaded at: https://www.mdpi.com/article/10.3390/cells13231953/s1; Figure S1. The knockdown of NMRAL1_Bovine and NMRAL2_Bovine and the effect of NMRAL1_Bovine knockdown on cell survival during *M. bovis* infection measured by the CCK8 Assay.
