# Supplementary material for: Bovine NMRAL2 Protein Blunts Nitric Oxide Production and Inflammatory Response in Mycobacterium bovis Infected Bovine Lung Epithelial Cells
Source: Cells. 2024 Nov 24;13(23):1953. doi: 10.3390/cells13231953 (PMC11640032; doi:10.3390/cells13231953)
Supplement: Supplementary file 1 [file cells-13-01953-s001.zip › cells-3318982-supplementary.pdf]

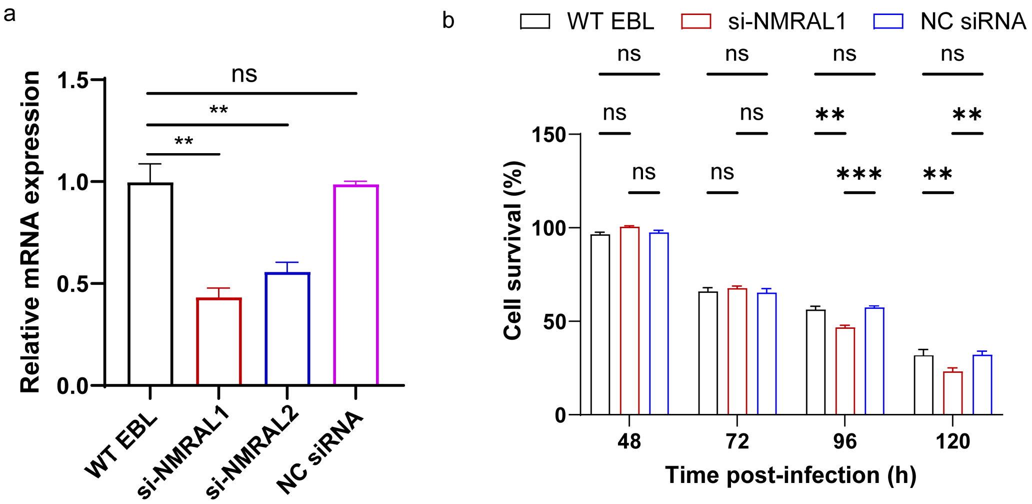

Figure S1. The knockdown of *NMRAL1* and *NMRAL2* and the effect of *NMRAL1* knockdown on cell survival during *M. bovis* infection measured by the CCK8 Assay. (**a**) WT EBL cells were transfected with siRNA targeting *NMRAL1* and *NMRAL2*, and transfection efficiency was confirmed via RT-qPCR 48 h post-transfection. (**b**) The impact of *NMRAL1* knockdown on cell viability was evaluated following *M. bovis* infection. Both WT and si-NMRAL1 EBL cells were infected with *M. bovis*, and cell survival was assessed using the CCK8 assay. Statistical significance between groups was determined using a two-tailed unpaired t-test and two-way ANOVA (three independent replicates). **$p < 0.01$ and ***$p < 0.001$ indicate statistically significant differences, and ns for no significant difference.
